# Challenges for the Post-Market Environmental Monitoring in the European Union Imposed by Novel Applications of Genetically Modified and Genome-Edited Organisms

**DOI:** 10.3390/biotech13020014

**Published:** 2024-05-15

**Authors:** Marion Dolezel, Andreas Lang, Anita Greiter, Marianne Miklau, Michael Eckerstorfer, Andreas Heissenberger, Eva Willée, Wiebke Züghart

**Affiliations:** 1 Land Use & Biosafety Unit, Umweltbundesamt–Environment Agency Austria (EAA), Spittelauer Laende 5, 1090 Vienna, Austria; anita.greiter@umweltbundesamt.at (A.G.); marianne.miklau@umweltbundesamt.at (M.M.); michael.eckerstorfer@umweltbundesamt.at (M.E.); andreas.heissenberger@umweltbundesamt.at (A.H.); 2 Büro Lang, Hoernlehof, Gresgen 108, 79669 Zell im Wiesental, Germany; lang@biologie.de; 3 Research Group Environmental Geosciences, Department of Environmental Sciences, University of Basel, Bernoullistr. 30, 4056 Basel, Switzerland; 4 Division of Terrestrial Monitoring, Federal Agency for Nature Conservation (BfN), Konstantinstr. 110, 53179 Bonn, Germanywiebke.zueghart@bfn.de (W.Z.)

**Keywords:** genome editing, genetic modification, new genomic techniques, European Union, post-market environmental monitoring

## Abstract

Information on the state of the environment is important to achieve the objectives of the European Green Deal, including the EU’s Biodiversity Strategy for 2030. The existing regulatory provisions for genetically modified organisms (GMOs) foresee an obligatory post-market environmental monitoring (PMEM) of potential adverse effects upon release into the environment. So far, GMO monitoring activities have focused on genetically modified crops. With the advent of new genomic techniques (NGT), novel GMO applications are being developed and may be released into a range of different, non-agricultural environments with potential implications for ecosystems and biodiversity. This challenges the current monitoring concepts and requires adaptation of existing monitoring programs to meet monitoring requirements. While the incorporation of existing biodiversity monitoring programs into GMO monitoring at the national level is important, additional monitoring activities will also be required. Using case examples, we highlight that monitoring requirements for novel GMO applications differ from those of GM crop plants previously authorized for commercial use in the European Union.

## 1. Introduction

The European Green Deal and its related policies aim to transform the EU society and economy to achieve carbon neutrality by 2050 [1]. The EU’s Biodiversity Strategy for 2030 highlights the importance of biodiversity protection as well as the recovery and restoration of nature to deal with the impacts of the climate crisis and the effects of the unsustainable use of natural resources. In the context of these EU targets, gathering environmental information and data are paramount to supporting the policy objectives and the impact assessment of specific policy measures, e.g., by using environmental indicators. Several EU policy areas increasingly rely on monitoring approaches for efficient impact assessment of environmental policies, e.g., soil health under the proposed soil monitoring law, the bioeconomy monitoring system, the climate monitoring mechanism, or biodiversity monitoring under the Nature Directives (Birds and Habitats Directives, Directive 79/409/EEC, and Council Directive 92/43/EEC). In addition, agri-environmental indicators are efficient tools for evaluating the effects of the EU’s Common Agricultural Policy (CAP) on environmental sustainability goals [2].

Genomic techniques, in particular transgenesis, have been used for many years to develop genetically modified organisms (GMOs) and specifically genetically modified (GM) crops. In the European Union, such GM crops are subject to authorization under the provisions of Directive 2001/18/EC and Directive (EU) 2018/350 for the deliberate release into the environment, as well as Regulation (EC) No. 1829/2003 and the Commission Implementing Regulation (EU) No. 503/2013 for food and feed use. These provisions require an environmental risk assessment (ERA), risk management measures, if appropriate, and post-market environmental monitoring (PMEM) for each authorized GMO or GM product [3]. So far, only crop plants have been authorized for commercial use in the European Union. The European Commission’s community register of genetically modified food and feed [4] lists all products subject to EC decision according to Regulation (EC) 1829/2003, including GM products withdrawn from the EU market. It includes GM cotton, maize, oilseed rape, soybean, sugar beet, and swede with herbicide tolerance and insect resistance as predominating GM traits, including stacking of these.

In recent years, novel genetic engineering tools based on the latest developments in molecular biology have been used for manipulating the genetic material of organisms. Particularly, the discovery of CRISPR/Cas (Clustered Regularly Interspaced Short Palindromic Repeats/CRISPR-associated protein), a nuclease-based genetic engineering tool, has simplified and accelerated the genetic modification of organisms, leading to a boost in research on genome-edited plants, animals, and microorganisms [5,6,7,8,9,10,11,12,13,14,15,16,17,18,19]. These new genomic techniques (NGT) have been successfully applied to other species than classical annual GM crop plants, such as horticultural crops [8,9], photosynthetic microorganisms including algae [15,17,18], trees [19], fish, and other organisms used for aquaculture [10] or livestock [12]. Novel traits such as tolerance to abiotic stressors, modified plant composition or morphology, and changed reproductive traits are the focus of research [20,21]. Such crops are increasingly being notified for experimental release in the EU according to Part B of Directive 2001/18/EC, e.g., drought-tolerant maize, salt- and drought-tolerant broccoli, and non-flowering tobacco plants [22,23]. However, none of these has so far been notified for commercial cultivation or for import for food and/or feed use. 

The objective of this study is to scrutinize existing concepts for GMO monitoring with regard to their applicability for novel types of GMOs, based on selected case studies. We show that novel GMO applications challenge PMEM in the EU. We demonstrate the need for improved monitoring by analyzing selected case examples of novel types of GMOs (GM microalgae, GM freshwater fish, and GM applications in fruit orchards) and simultaneously highlighting specific monitoring requirements. The targeted traits in these GMOs were either genetically modified by transgenesis or genome-edited by targeted mutagenesis (e.g., CRISPR/Cas). According to a ruling of the Court of Justice of the European Union in 2018, techniques for targeted mutagenesis are subject to the provisions of Directive 2001/18/EC [24]. Consequently, the term “genetically modified” (GM) will be used for all examples, independent of the technique used to modify or edit the genetic makeup of the respective organism. In June 2023, the European Commission (EC) published a proposal for a new regulation that would exempt plants produced by certain genomic techniques from the current GMO regulations [25]. However, it is uncertain at present if the proposed regulatory changes will be implemented. The EC approach is controversially discussed and considered not to be adequate [26]. The changes proposed by the EC would also not apply to organisms such as animals, microorganisms, and viruses obtained by NGT. 

In accordance with the requirements of Annex II of Directive 2001/18/EC, we outline potentially adverse environmental effects for each case study resulting, e.g., from the spread of the GMO in the environment, the transfer of the inserted genetic material to other organisms, phenotypic and genetic instability, interactions with other organisms, and changes in management, including agricultural practices. Based on the results of these assessments, specific monitoring requirements are identified and crosschecked with GMO monitoring concepts and guidelines currently available. In addition, we outline existing nationwide biodiversity monitoring programs, such as those available in Germany, and show that these can play an important role in general surveillance of novel GMO applications. We conclude that additional surveillance activities will have to be included in PMEM to detect potential adverse effects of such applications on the environment.

## 2. Concepts and Guidelines for PMEM

In the EU, the monitoring of potential adverse effects of GMOs on human health and the environment after authorization for import or cultivation is obligatory [3,27]. As part of the authorization decision, the applications must contain a post-market environmental monitoring plan. The monitoring provisions, according to Annex VII of Directive 2001/18/EC distinguish between case-specific monitoring and general surveillance. While the former intends to confirm the assumptions made in the ERA with regard to direct and indirect environmental effects, the latter aims to detect long-term effects and effects not foreseen in the ERA. So far, monitoring plans have been developed and submitted for annual GM crop plants only (e.g., maize, oilseed rape, or soybean). To detect unanticipated environmental adverse effects of GMOs in the context of general surveillance, Annex VII of Directive 2001/18/EC, foresees the possibility of making use of existing environmental surveillance networks [3]. However, for the only GM crop currently cultivated in the EU (insect-resistant maize MON810), no such programs have been used so far, and general surveillance is mainly based on farmer questionnaires [28,29]. 

Given that these monitoring plans are not sufficiently adequate to detect adverse effects on biodiversity in the EU [30,31], and with the aim of promoting the implementation of PMEM in Germany and other EU member states, a range of GMO monitoring concepts or methodological guidelines have been developed on behalf of or supported by the Federal Agency for Nature Conservation since 2005. These publications comprise research reports, scientific publications, and technical and methodological guidelines, and, to a lesser extent, conference reports, proceedings, or policy documents. A compilation of all publications can be found in [32]. 

These GMO monitoring concepts or methodological guidelines generally refer to the total area of Germany and were mainly developed for currently authorized GM crops (e.g., GM maize, GM oilseed rape). Some of the publications address conceptual approaches and general implementation aspects of GMO monitoring, such as the selection of relevant monitoring sites, monitoring methods, and indicators (e.g., [31,33]). Zünd et al. [34] refer to the monitoring of the spontaneous occurrence of GM plant populations in the environment. The studies of Meier and Hilbeck [35] and Hilbeck et al. [36,37] address the definition of criteria when selecting indicator species for GM maize and GM oilseed rape. Hilbeck et al. [38] develop a concept for a selection matrix for aquatic indicators for monitoring aquatic biodiversity. Lang et al. [39], Römbke et al. [40], as well as Sudfeldt and Trautmann [41], discuss the usefulness of butterfly, soil, and bird monitoring for the monitoring of GM crops in an agricultural context. The study of Wedlich et al. [42] discusses the selection and determination of the monitoring area and period as well as sampling for monitoring of GM oilseed rape during import. Other studies [43,44] describe the exposition of aquatic ecosystems when cultivating *Bacillus thuringiensis* (*Bt*) maize, including suggestions for toxicity testing of selected water organisms. 

In addition, guidelines have been developed in collaboration with the Association of German Engineers (VDI) to provide standardized principles and methods for GMO monitoring [45,46]. The VDI guidelines comprise monitoring methods for wild bees [47], ferns and flowering plants [48], butterflies and moths [49], pollen sampling [50,51], amphibians [52], and soil organisms [53]. Methods for floristic mapping of genetically modified plants, their crossing partners, and their hybrid offspring are also available [54]. In addition, guidelines are available that address sampling of plant material for molecular biology analysis [55], detection of nucleic acids and *Bt* proteins in the environment [56,57].

These GMO monitoring concepts and guidelines have not been implemented in practice thus far. The question of whether the currently applied monitoring approaches are suitable for novel types of GMOs other than annual crops has not been addressed and verified by the respective authorities yet.

## 3. Biodiversity Monitoring Programs in Germany

In Germany, several biodiversity monitoring programs on the national level have been developed or are currently under development and are coordinated by the Federal Agency for Nature Conservation. These include bird monitoring, High Nature Value (HNV) farmland monitoring, Fauna and Flora Habitat (FFH) monitoring, insect monitoring, ecosystem monitoring, and the monitoring of national heritage (for an overview see [32]). 

Bird monitoring in Germany monitors common and rare breeding birds, as well as resting waterfowl outside the breeding period [58,59,60,61]. Standardized methods and established monitoring structures ensure the collection of long data series. 

High Nature Value (HNV) farmland monitoring in Germany assesses the status and changes of biological diversity within the agricultural landscape, focusing on agricultural areas with high nature value, such as extensive land use, semi-natural vegetation types, highly structured land use, or the occurrence of rare and specialized fauna and flora [62,63]. 

The Fauna-Flora-Habitat (FFH) monitoring in Germany is based on Article 11 of Directive 92/43/EEC, which requires EU Member States to monitor the conservation status of certain species and habitat types (Annex I, II, IV, and V). The monitoring shall provide data in order to assess their conservation status [64,65,66]. Data collection and analysis of the FFH monitoring follows a systematic and standardized method, thus providing robust scientific results. 

Insect monitoring is currently under development in Germany, covering common and rare as well as protected insects using standardized methodological guidelines [67,68,69]. 

Ecosystem monitoring is also under development in Germany, continuously surveying and assessing biotope types and the state of ecosystems in nationwide representative sampling areas [70,71,72]. These specific areas are already used in the context of HNV farmland monitoring, the monitoring of common breeding birds, and insect monitoring. Major drivers of biodiversity loss, such as land use change, intensification, or climate change, will also be assessed. 

The National Heritage in Germany comprises areas of conservation concern owned by the Federal Government of Germany. Among others, it covers areas formerly used for military purposes, mostly in northern and eastern Germany (e.g., the “green belt”), which comprise mostly forests. First National Heritage monitoring schemes have been developed, such as forest monitoring and simplified monitoring of breeding birds and butterflies [73,74]. 

## 4. Monitoring Requirements of Novel GMO Applications

### 4.1. Example 1: GM Applications in Fruit Orchards

Transgenic and genome editing approaches are being used in a range of different horticultural crops, mainly vegetables but also fruit trees (see an overview in [8]). To our knowledge, the only GM horticultural crop that has received market approval worldwide is a transgenic apple [75]. In the EU, no applications of GM horticultural crops have been authorized for market application so far, but field trials with cisgenic apple trees have been conducted, e.g., in the Netherlands and Switzerland [76,77].

Traits targeted by genetic modification are the acceleration of the breeding cycle, modified fruit quality, and particularly disease resistance, since apple trees are affected by many economically important diseases [19,78]. The biotechnological approaches aim at knocking out plant susceptibility factors for bacterial or fungal pathogens or expressing resistance genes by cisgenesis or transgrafting [20]. An example is a cisgenic apple with increased resistance to apple scab containing a resistance gene from *Malus floribunda* [78,79,80]. Genome-edited apple trees were also developed using CRISPR/Cas-induced gene knockouts of susceptibility genes to increase apple resistance to fire blight [81,82].

In addition, GM virus approaches are applied to horticultural crops to target bacterial disease agents. The bacterium *Candidatus Liberibacter asiaticus* causes the Citrus Greening Disease, also known as Huanglongbing, in citrus and orange trees [83]. The disease and its corresponding vector species, psyllids, have spread from Asia to the US, where they cause dramatic damage to the commercial orange and citrus fruit industry, e.g., in Florida [84]. So far, in Europe, no damage has been reported on commercial citrus fruit plantations [85], and the bacterial pathogen is listed as a quarantine pest [86]. In the US, a genetically modified viral vector, the GM Citrus Tristeza Virus (CTV-SoD), has been developed as a potential remedy. The GM virus expresses antimicrobial proteins (defensins, derived from spinach) when infecting adult citrus trees, e.g., via inoculated scions grafted onto tree stocks [87]. In Florida, the GM CTV-SoD virus has already been tested in small-scale field trials. Further larger-scale releases were proposed by the Southern Gardens Company in 2020 but have not been conducted yet [88]. 

In its pest risk assessment and the environmental impact statement, the respective US regulatory authority discussed the potential adverse environmental effects of the GM virus (CTV-SoD) [87]. The authority evaluated whether the release of the GM virus would result in novel risks to those already presented by CTV strains naturally occurring in the US. In addition, two other studies reviewed the GM CTV-SoD application: EFSA discussed the CTV-SoD virus application as a case study in the framework of an opinion analyzing different microorganisms derived from synthetic biology [89]. Another recent study reviewed environmental applications of GM viruses, including the GM CTV-SoD application, as an example of a GM virus used to treat a bacterial plant disease [88]. Both studies noted that the assessment conducted by the USDA did not cover all the risk areas that would need to be considered in an environmental risk assessment in the EU under Directive 2001/18/EC.

#### Potential Environmental Effects and Consequences for Monitoring

In contrast to crops, which are annually released into fields and harvested after the cropping season, fruit trees can vegetatively reproduce and disperse over long periods of time and over large spatial scales once planted in an orchard. Fruit trees have a long lifespan of approximately 15 years in plantations but several decades in nature [78]. 

Although the focus of GM horticultural applications will mainly be in commercial plantations and orchards, use in public spaces (e.g., edible cities) and private orchards and gardens cannot be excluded. In addition to commercial trade, the sale or exchange of fruit trees to and between private individuals (e.g., via cuttings or scions) is also an important means of dispersal of plant material. Monitoring efforts have to focus not only on the commercial use of GM fruit trees but also on private and public use, as well as on semi-natural and natural habitats, including woodland. 

The establishment of GM trees or GM hybrids in natural habitats, as well as the outcrossing of a GM trait into a species of conservation concern, could result in legal and practical nature conservation issues [90]. For example, the European crab apple (*Malus sylvestris*) is included in the Red List of some EU countries, such as Germany [91,92]. The presence of the GM trait could threaten the legal status of the protected wild-type species. In addition, the establishment of GM trees in natural habitats poses questions regarding their naturalness [93].

The outcrossing of the GM trait (e.g., fire-blight resistance) to wild relatives, e.g., in the case of GM apple trees, can occur via pollinators (e.g., honeybees, bumblebees, or wild bees), although wind pollination is also possible. Hybridization of cultivated apples has been described with wild apple *Malus sylvestris* and other *Malus* species, as well as with *Pyrus* sp. and *Sorbus* sp. [94,95]. The progeny of GM apple trees is mostly found at a distance of 5–10 m from the pollen donor, but to a lesser extent also at a distance of 100 m [96]. The fire-blight resistance trait may provide a fitness benefit to wild taxa, thereby leading to changes in plant composition in the respective habitats. However, fire blight resistance already occurs naturally in wild apples, and therefore this GM trait is unlikely to contribute to an expansion of the habitat of resistant apple trees.

For a GM virus application in orchards such as the CTV-SoD, all potential host plants are relevant. If the GM virus can be dispersed by infected plant material or vector insects, it could spread to other (wild) citrus plants. Many fruit trees can spread into semi-natural or natural habitats by pollen and seeds, but also by vegetative dispersal (e.g., root suckers). It is unknown whether seed, fruit, or vector insects like aphids can disperse the GM virus under European conditions [89]. So far, it remains unclear whether the GM virus is restricted to the cultivation areas of fruit trees (orchards) inoculated with GM virus-infected scions, or if the exposure could extend outside these horticultural areas.

In Europe, citrus plants, which could also serve as a potential host for CTV, are lemon, lime, orange, mandarin orange, and grapefruit, including different hybrids. The potentially exposed environments in the EU are largely restricted to the warmer regions of Europe, such as the Mediterranean region, where these plants are cultivated or thrive in natural habitats. Among potential hosts, citrus rootstocks, e.g., the trifoliate orange (*Poncirus trifoliata*) or ornamental citrus plants such as the calamondin orange (*Citrofortunella microcarpa*), may also be relevant [89]. 

Adverse effects on host plants may occur through unintended changes in the infectivity and pathogenicity of the GM virus [87]. If the bacterial pathogens develop resistance to the defensins, the efficacy of the GM virus application can decrease. Similarly, for the GM fire blight resistant apple, a resistance breakthrough of the pathogen-causing fire-blight bacterium *Erwinia amylovora* may lead to re-infections, with implications for additional plant protection measures. Resistance breakthroughs may also affect species or varieties with naturally existing resistance (e.g., old varieties). Fire-blight resistance has already been overcome by some *Erwinia* strains in North America and Israel [97]. For the timely detection of a potential resistance breakthrough in the target organisms, data on the incidence of the disease need to be available for monitoring. 

Sustained suppression or elimination of the targeted pathogen may change the pathogen spectrum, with other (non-target) pathogens occupying the niche of *E*. *amylovora*. The occurrence and incidence of other (non-target) pests and pathogens in commercial orchards, including the use of pesticides, should therefore be monitored to detect any shifts in the pathogen spectrum in the case of a GM horticultural application with a resistance trait. The antimicrobial product expressed by the GM virus (the defensin) may impact not only the target organism (the bacterial pathogen) but also other potential microbial pathogens such as Gram-negative and Gram-positive bacteria or fungi [87,98].

In the commercial context, disease-resistant (e.g., fire-blight-resistant) fruit tree varieties may represent a significant economic advantage, leading to enlargement of the cultivation area. Most commercial apple plantations are biodiversity-poor, intensively managed with corresponding applications of fertilizers and plant protection products against diseases, pests, or weeds [99]. Adverse effects on biodiversity may therefore increase, such as the impacts of commercially used pollinators on wild bee populations [100,101,102] or indirect effects on non-target organisms, biodiversity, or organically cultivated orchards [103,104]. 

Knowledge of the potential adverse environmental effects of a GM virus application in fruit orchards is still limited, mainly due to the lack of knowledge on the biology and ecology of the insect vectors of CTV viruses present in Europe. In addition, knowledge about non-target organisms in citrus orchards under EU conditions is currently very incomplete. Information from risk assessments and field trials carried out in non-EU regions may help fill existing knowledge gaps, but it cannot replace gathering relevant data specific to the specific receiving environments in Europe. The monitoring requirements for GM applications in orchards therefore mainly comprise virus dispersal to potential host trees, plant health, and monitoring of effects on biodiversity (Table 1).

### 4.2. Example 2: GM Freshwater Fish

GM technology, including genome editing, is extensively used for the genetic modification of several marine and freshwater fish species [10,105], with more than 20 species used in aquaculture [106,107]. GM applications of fish focus on growth performance, reproduction and development, disease resistance, pigmentation, and product quality [107,108]. GM fish, such as growth-enhanced salmon [109], have received market approval in the US and Canada, and various species of aquarium fish are available in different fluorescent colors in the US and Canada [110]. In Argentina and Brazil, gene-edited growth-enhanced Nile tilapia (*Oreochromis niloticus*) has been exempted from GMO regulation [108]. In Japan, genome-edited Red Sea bream and tiger puff that grow bigger than their conventional counterparts have been notified for commercial sale recently, without regulatory oversight and risk assessment [111]. In the European Union, no application for import, release, or commercial use of GM fish has been submitted so far. However, in Norway, a GM salmon with a sterility trait has been risk-assessed for use in field trials [112]. Guidance for environmental risk assessment of GM animals published by EFSA also covers GM fish [113].

Of the hitherto modified GM freshwater fish species, carp species such as common carp (*Cyprinus carpio*), grass carp (*Ctenopharyngodon idella*), gibel carp (*Carassius gibelio*), and rainbow trout (*Oncorhynchus mykiss*) are of relevance to the EU. For these species, research currently focuses on the following applications: growth enhancement or improved muscle growth [114,115,116], sterility or sex reversal [117], disease resistance [118], and pigmentation [119]. Due to the market relevance, the following considerations focus on GM carp and rainbow trout with growth enhancement traits. These traits can be achieved, e.g., by disrupting the mstnba gene that encodes myostatin using CRISPR/Cas. Myostatin is a growth factor that inhibits muscle cell growth; hence, its inhibition leads to increased muscle fiber number and size (muscle growth), body weight and fish length [114,120]. Genome editing was also used to improve growth and feed conversion efficiency in gibel carp by targeting a lipid kinase gene [116].

#### Potential Environmental Effects and Consequences for Monitoring

Fish are mobile animals that grow over several years in fish farms, ponds, and other aquaculture facilities. Therefore, not only intentional releases must be considered, but also their potential for unintentional escape into natural water bodies. Escaped or accidentally released GM freshwater fish can establish and persist in those water bodies that meet their habitat requirements. This has been shown by GM fluorescent zebrafish, which escaped from production ponds in Brazil [121]. The temperature regimes necessary for growth and reproduction are highly species-dependent and are likely to be similar between GM growth-enhanced freshwater fish and non-GM fish. The life expectancy of carp and rainbow trout is 25–30 and 3–8 years, respectively [122]. If GM growth-enhanced fish are introduced into natural habitats, their potential for spread and persistence over a long period of time must be considered. Both carp and rainbow trout are predators in aquatic habitats that affect prey communities through top-down control, although the likely effects depend on their specific role and resource partitioning with other species present in the respective habitat [123].

The exposure to GM freshwater fish will occur mainly through fish farming in artificial tanks, natural ponds, or other types of aquacultures. Carp is mainly kept in warmer ponds with low water flow rates, often in polyculture with other fish species, while rainbow trout is raised in colder water, often in artificial tanks, but also in ponds with higher flow rates [124,125]. Due to their larger body size, GM growth-enhanced fish are likely to be of interest to recreational fisheries (e.g., angling), both commercially and privately. The GM trait in these fish species could also incentivize illegal stocking or use in private ponds. Besides taste, trophy quality is a reason for introducing and stocking certain angling species [126] and fish size is important for the anglers’ satisfaction [127].

For non-GM fish, accidental releases from experimental or aquaculture facilities, including hatcheries, or during transport are documented, leading to the presence of non-native or genetically distinct fish populations in diverse aquatic habitats. In the EU, domesticated common carp used for aquaculture and recreational fisheries has become feral or even invasive in natural habitats in a range of EU countries [128]. For other fish species, the abundance of escaped farmed fish in rivers correlates with aquaculture intensity [129]. Such accidental releases occur frequently with (non-GM)-cultured salmon from marine fish farms [130]. Also, escapes of cultured freshwater fish like the rainbow trout from fishponds are known [125,130,131]. Extreme weather events like floods can enable the escape of fish from inland aquaculture facilities, as these are generally connected with natural water bodies for freshwater supply and water runoff. A basic requirement for monitoring is therefore the surveillance of the number and frequency of released GM fish into aquaculture facilities or natural habitats (e.g., ponds used for angling; Table 2). 

A major environmental risk of GM growth-enhanced fish is the potential hybridization of fish that escaped from aquacultures with wild progenitors and the possible resulting population effects, such as the dispersal of transgenes and changes in the genetic makeup of native fish populations, habitat alteration, and food web effects [132]. For freshwater fish in the EU, this concerns the potential hybridization between GM rainbow trout and autochthonous trout populations, which are important protection goals (e.g., brown trout). Even though rainbow trout rarely spawn in Europe, self-sustaining populations are known, and interspecific hybridization of rainbow trout with brown trout is possible. Adverse impacts have already been documented from escaped or introduced (non-GM) trout populations on local native brown trout populations and freshwater habitats [133]. Also, for wild common carp stocks, hybridization with introduced stocks is considered a threat [134,135]. GM fish with growth enhancement may show earlier sexual maturity or produce a higher number of eggs. Moreover, if GM growth-enhanced fish have a competitive mating advantage but at the same time reduced offspring survival, fish populations may be suppressed, displaced, or even driven to extinction (Trojan gene effect). Increased competitive behavior of growth-enhanced fish, e.g., in terms of occupying suitable sites for oviposition or feeding, is also conceivable (see [105] and references therein).

In transgenic, growth-enhanced salmon, foraging rates, feeding motivation, and dominance are higher than in non-GM fish [136]. Exact data on the feeding behavior of other GM-growth-enhanced fish species (e.g., GM carp) are lacking so far, but there are indications that the feeding motivation of GM carp is also considerably higher than in non-GM fish [137,138]. Increased food resource acquisition by GM growth-enhanced fish can affect the composition and structure of the aquatic species community in a given water body under natural conditions. Due to competitive advantages over non-GM and smaller fish, GM growth-enhanced carp or rainbow trout may suppress or even displace native fish from their habitat [139,140]. In the case of rainbow trout, cannibalistic behavior may increase with increased appetite. However, under natural conditions, trade-offs with other (unintended) traits that counteract such positive fitness-related traits, e.g., changes in predator avoidance, are also possible [105].

Monitoring activities should therefore focus on the frequency of occurrence of GM growth-enhanced fish in natural and semi-natural habitats, as well as the potential effects of the GM fish on the native fish and invertebrate community. Effects on the quality of aquatic habitats may occur due to the churning up of sediment, damaging aquatic macrophytes, or water eutrophication. Such alterations in habitat quality can also affect higher trophic levels, such as amphibians and birds [141,142,143]. 

Any further intensification of aquaculture production due to GM growth-enhanced fish may result in an aggravation of adverse environmental effects that are already evident for conventional freshwater fish farming, such as the entry of organic pollutants, the discharge of pathogens and parasites, or the use of pharmaceuticals and disinfectants [144,145]. Consequently, the surveillance of a potential intensification of freshwater fish production and the spread of diseases, parasites, and pathogens to native fish populations is required.

### 4.3. Example 3: GM Microalgae

Microalgae are microscopic, mostly unicellular, photosynthetic algae found in either marine or freshwater habitats. Microalgae have the ability to fix atmospheric CO_2_ and produce different industrially relevant substances and raw materials of uniform quality without claiming arable land [146]. In recent years, microalgae have been of scientific interest for the production of renewable fuels or functional foods, i.e., nutraceuticals, and feed for aquaculture [147,148]. In the European Union, the commercial use of (non-GM) microalgae is currently at its beginning. Environmental applications of GM microalgae are not yet commercially available. However, research is ongoing, both for genetically modified and genome-edited taxa.

Transgenic microalgae are currently being utilized for the production of industrial enzymes, pigments, fatty acids, lipids, therapeutic proteins, including vaccines, biofuels, high-quality food additives, or feed for aquaculture purposes, although the production of recombinant proteins in microalgae is not yet cost-effective [17]. About 100 species of microalgae have been modified using transgenesis (see overview in [17,149]). Transgenic *Nannochloropsis oculata* expressing fish growth hormones are fed to brine shrimps (*Artemia* sp.), which in turn are fed to fish larvae in aquaculture (e.g., *Tilapia* sp.), resulting in higher growth rates in farmed fish [150]. Australia has approved the controlled release of the marine species *Nannochloropsis oceanica*, expressing increased fatty acid levels. In covered production facilities, the GM algae were tested until 2023 [151]. In the US, the green algae *Scenedesmus dimorphus* was cultivated in small open ponds in order to study its potential for dissemination and spread into the environment and its potential ecological effects [152]. The genetic modification involved the production of fatty acids as well as marker genes for detection purposes. In addition to these transgenic approaches, a range of mutagenesis techniques, such as zinc-finger nucleases, TALEN, and CRISPR/Cas, have been used for genome editing in microalgae [15,18,153]. 

One of the relevant taxa for genome editing is *Chlamydomonas* sp., a genus of flagellated green algae with more than 580 species, mostly occurring in freshwater but also in the sea, soil, ponds, eutrophic lakes, and snow [154]. Jiang et al. [155] showed for the first time the application of the CRISPR/Cas-system in *C*. *reinhardtii*. Also, other authors achieved genome editing in this species by using CRISPR/Cas [156]. They used targeted two-gene knockout by CRISPR Cas9, generating a strain that over-expressed zeaxanthin for improved photosynthesis under high-light conditions [156]. Another important genus is *Nannochloropsis* sp., an immobile microalga with several morphologically non-distinguishable species, mostly occurring in marine environments but also in freshwater and brackish water [157]. This genus has the ability to form pigments in high concentrations, such as astaxanthin, zeaxanthin, or canthaxanthin [158]. Its particular industrial relevance is due to its ability to accumulate polyunsaturated fatty acids. It is also considered promising for the production of biofuels [159]. Ajjawi et al. [160] attenuated the expression of a fatty acid regulator (ZnCys) in *N*. *gaditana* by use of a combined CRISPR/Cas9-RNAi mechanism, leading to an increased production of triglycerides (Triacyl-Glycerol TAG) with concurrent high productivity [160].

#### Potential Environmental Effects and Consequences for Monitoring

Due to their small size (e.g., 10 μm for *Chlamydomonas* sp. and 2–5 μm for *Nannochloropsis* sp.), the unintended dispersal of microalgae into natural habitats can occur via a range of different pathways along the production chain, such as during cultivation, harvest, processing, disposal, or use of the final product. High-volume production of microalgae is predicted to take place mostly in open facilities, such as raceway ponds [161]. Consequently, monitoring the spread and occurrence of GM microalgae in natural aquatic habitats is crucial. Microalgae are dispersed by air and aerosols, spillage, flooding, or even by different animal and human vectors. Due to the necessary mechanical mixture of the algal suspension during production in open ponds, aerosol formation and dispersal by air cannot be prevented. Wilkinson et al. [162] showed in modelling studies that the dispersal of microorganisms smaller than 9 μm via air can occur over several days and large distances, depending on air temperature, humidity, and the susceptibility for desiccation of the respective species. Szyjka et al. [163] experimentally showed the dispersal of GM algae by air over a distance of at least 50 m from an open facility. During the harvest of the algal biomass, microalgae can enter the environment via drainage water. When cultivating GM microalgae in closed facilities, accidental release may occur due to leakage from bioreactors. In addition, the transport of algal suspensions or dried algae can pose an exposure risk if the GM algae are still viable. In addition, extreme weather events may also contribute to the accidental release of microalgae. 

Microalgae thrive in a range of natural freshwater habitats, such as lakes, ponds and puddles, watercourses, ditches, streams, and rivers, as well as brackish and seawater habitats. The persistence and survival of GM microalgae in natural habitats will depend on the habitat requirements of the respective species as well as on their ability to tolerate adverse (abiotic and biotic) environmental conditions. It is unknown whether *Nannochloropsis oceanica*, in general a marine species, can also survive in freshwater or rainwater. Szyjka et al. [163] showed that cultivated GM freshwater algae (*Acutodesmus dimorphus*) were able to survive in natural freshwater habitats. The survival of GM microalgae will also depend on the GM trait(s) and their effect on the fitness of the species in their respective habitats. However, predictions of the risks of introduced GM algae (or non-native algae) in natural habitats based on laboratory experiments or modelling studies are considered very difficult, if not impossible [164]. In addition, the possibility of dormancy of microalgae cells under dry conditions has to be taken into consideration. Therefore, their survivability may be extended for long periods of time. Vertical gene transfer plays a role for algal species with a sexual reproduction cycle if wild-type algae of the same taxon are present in the environment. Many species of microalgae reproduce mainly or exclusively (e.g., *N*. *oceanica*) asexually, but can switch to sexual reproduction, depending on the prevailing environmental conditions.

Microalgae form the basis of aquatic ecosystems and food webs. GM microalgae with increased productivity of fatty acids such as TAG (Triacyl-Glycerol) for biofuel production may affect the entire aquatic food web. The specific effect depends on the specific fatty acid that is targeted by the genetic modification [151]. For example, toxic substances or a reduced food quality for higher trophic levels (e.g., predators) may occur due to biochemical changes. Fatty acids are important for the structural integrity and stability of cell membranes in microalgae [165]. Changes in the composition of the cell membrane can therefore induce growth or fitness differences between GM and wild-type microalgae. Fatty acids are also released by microalgae into the environment, e.g., during stress conditions or cell damage and lysis, e.g., after algal blooms. Some fatty acids have antimicrobial effects, serving as protection for microalgae. Changes in the fatty acid composition of microalgae may entail a suboptimal food composition for predators and/or pathogens and significantly affect zooplankton populations, i.e., grazers. The C:N and C:P ratios, respectively, as well as long-chain, polyunsaturated fatty acids, are essential for the nutritional value of microalgae for copepods [166]. The specific composition of lipids is also important for the early larval stages of mussels [161]. A decrease in predation rates and increases in the competitive ability of GM microalgae in natural habitats could lead to algal blooms with consequences for the aquatic ecosystem and related ecosystem services, such as the provision of food through fisheries. Such risks are particularly relevant for algal taxa that are known to be toxin-producing species or high-biomass producers. Flynn et al. [167] extensively discussed the implications of biofuel-producing GM microalgae on predator-prey relationships.

The ecological importance of microalgae as the basis of aquatic food webs justifies a rigorous monitoring effort when cultivating GM microalgae, as well as constant surveillance of potential adverse effects in natural aquatic habitats. Algal communities in natural habitats are complex and highly dynamic. This complicates the detection, assessment, and monitoring of the environmental effects of GM microalgae in cases of spread and establishment in natural habitats. Monitoring of the aquatic food web as well as of keynote species will be needed, focusing on the effects on ecosystem functions in natural aquatic habitats. Potential adverse effects on specific protection goals, e.g., ecosystem services, in aquatic habitats also need to be considered (Table 3). In this context, the pre-release assessment of the baseline condition in potential receiving environments is particularly crucial.

## 5. Discussion and Recommendations

### 5.1. Limitations of GMO Monitoring Concepts and Guidelines for PMEM for Novel GMO Applications

**Figure 1:**
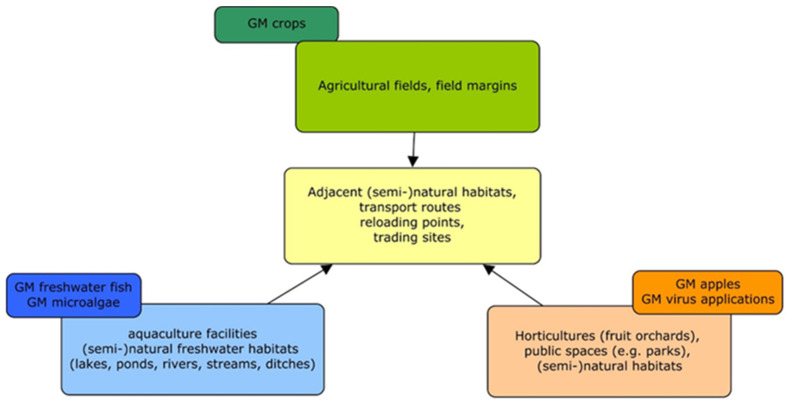
Receiving environments of novel GMO applications compared to GM crops (own drawing).

The first GMO applications in the EU comprised mainly GM crops, such as *Bt* maize in Spain [168]. Therefore, the GMO monitoring concepts or methodological guidelines developed so far consider crop plants grown in agro-environments (e.g., GM maize, GM oilseed rape) rather than other receiving environments, which may be affected by novel GMO applications (Figure 1). However, some conceptual approaches and general implementation aspects of GMO monitoring, such as those outlined by Züghart et al. [31,33], can also be used for monitoring the effects of novel GMO applications addressed in this study. The conceptual study by Zünd et al. [34] considers the import of GM oilseed rape as a case study. The conceptual approach to designing a monitoring strategy, in particular regarding the determination of exposure and selection of monitoring areas, can also be applied to other types of GMOs. However, taxon-specific adaptations would be required, e.g., in the case of the cultivation of GM apple trees or the release of GM animals in aquatic environments. The general procedure of criteria selection for indicator species addressed by Meier and Hilbeck [35] and Hilbeck et al. [36,37] is also applicable to other types of GMOs. The faunistic survey methodologies, survey designs, baselines, reference areas, and suggested indicator groups (birds, butterflies, and soil organisms) discussed in Lang et al. [39], Römbke et al. [40], and Sudfeldt and Trautmann [41] can be used in monitoring studies as well as in the case studies discussed here. As the focus of these three studies is on agricultural habitats, specific adaptations will be required if applied to other terrestrial (non-agricultural) ecosystems. Similar limitations are relevant to the study of Wedlich et al. [42]; however, their concept to define monitoring areas and to detect feral populations can also be applied to novel GMO applications, e.g., GM trees, in terrestrial habitats.

The basic principles and strategies for monitoring outlined in the VDI guidelines [46] describe the general procedure, concept, and design of GMO monitoring, which is also of value for other GMO applications. All other guidelines describe specific methodologies for monitoring faunistic taxa, plant communities, soil organisms, or pollen sampling. As all VDI guidelines focus on the effects of GMOs grown in agro-environments, the protocols might have to be adapted on a case-by-case basis, e.g., if other areas are to be monitored (e.g., orchards, parks, transport routes).

VDI guidelines covering particular plant and animal species are also useful for monitoring novel GMO applications, if the respective taxa are selected as relevant indicators for a particular application. For example, the VDI guidelines that cover wild bees [47], diversity of flowering plants [48], or butterflies [49] are applicable for monitoring biodiversity in apple tree orchards. However, pollinators other than wild bees and butterflies have not been taken into consideration and would have to be complemented, if considered relevant. 

The VDI guidelines for pollen sampling [50,51] are applicable if adapted for monitoring the spread of pollen other than maize pollen, e.g., pollen of apple or fruit trees. 

The VDI guidelines for the preparation of plant samples and PCR methods [55,56] are likely of limited value for novel GMO applications due to the methodological developments in the past years. The guideline on the immunochemical detection of insecticidal *Bt* proteins cannot be applied to the case studies discussed as the focus is on the detection of *Bt* proteins expressed in GM maize [57]. 

Concepts and guidelines relevant for monitoring GMOs in aquatic habitats are rare. The matrix provided by Hilbeck et al. [38] for selecting aquatic indicators is generally applicable also for GM fish or GM microalgae; however, the selected taxa refer to the introduction of GM plant material into a specific type of running water and would therefore have to be adapted to the specific needs of the respective GMO application. Other studies [43,44] focus on the identification of aquatic habitats that are relevant for the introduction of GM maize residues, but the selection procedure for exposed aquatic habitats as outlined in their studies may be useful for GM applications in aquatic habitats. Only one of the VDI guidelines covers (semi-)aquatic organisms (amphibians, [52]). This guideline also focuses on agro-environments, with limited relevance for monitoring the effects of GM freshwater fish or GM microalgae.

### 5.2. Complement Existing Biodiversity Monitoring Programs for Monitoring the Effects of Novel GMO Applications

The analyzed biodiversity monitoring programs in Germany are useful for monitoring the effects of novel types of GMOs as they can provide baseline information on the state of the environment, particularly for the monitoring of long-term and indirect effects on biodiversity. However, these national programs can only partially meet some of the novel monitoring requirements identified in this study (Table 4 and Table 5). 

The existing bird monitoring programs in Germany can be used to survey and detect possible long-term, indirect, and cascading effects of novel GMO applications as birds represent a higher trophic level within the food web. This may be relevant for detecting intensification effects in GM horticultures, loss of biodiversity, or pesticide use in GM orchards, but also for detecting effects on higher trophic levels in aquatic communities incurred by the use and production of GM fish or GM microalgae. The German bird monitoring generally covers environments relevant for the discussed case studies of novel GMO applications, although an increase in sample size when monitoring certain areas and bird species may be necessary. In addition, it provides baseline data to enable a comparison with the situation after the release of any kind of GMO.

The HNV farmland monitoring in Germany may be suitable for analyzing the indirect effects of novel GMO applications at the landscape scale, such as detecting a decrease in HNV farmland area due to, e.g., intensification of production. HNV farmland monitoring will be useful if combined with other monitoring programs at the same sites, such as bird monitoring or ecosystem monitoring. However, for monitoring effects at the species level, data resolution is not sufficient. 

FFH monitoring can be an important part of GMO monitoring if novel GMO applications affect protected habitats or protected species covered by this monitoring. Protection goals covered by FFH monitoring may be adversely affected, especially if mobile GMOs (e.g., GM fish) are concerned or if spatial proximity to habitats where GMOs are released is evident. FFH monitoring could support the monitoring of the occurrence of feral GM apple trees or wild relatives of conservation concern (e.g., *Malus sylvestris*) when monitoring plant communities in protected habitats. Likewise, the occurrence of GM fish or the displacement of native and autochthonous (and protected) fish species (e.g., Brown trout, *Salmo trutta fario*) in protected habitats can be integrated into this monitoring program. The monitoring frequency and sample sizes used in FFH monitoring may not be sufficient and should be increased, as effects on specific protected habitats must be detected as early as possible. 

Insect monitoring in Germany can be a valuable tool for the monitoring of novel GMO applications, as it covers habitat types of the overall landscape, including farmland, grassland, woodland, and residential areas (depending on the insect group). The insect monitoring is suitable to survey possible effects on pollinators and flower-visiting insects, e.g., wild bees, butterflies, or hoverflies in orchards. Insect monitoring can survey changes in the composition and/or structure of aquatic invertebrate communities, which could be useful for monitoring possible effects on water insects due to GM fish and GM microalgae. In addition, it can indicate indirect effects, e.g., adverse effects on insects through pesticide use, e.g., in GM applications in orchards.

To monitor the effects of novel types of GMOs, ecosystem monitoring will be useful when complemented with the results of bird monitoring, HNV farmland monitoring, and insect monitoring. It will supply data concerning land use and habitat types within the broader landscape, which complements the monitoring of protected or endangered habitats and species covered by FFH monitoring. The ecosystem monitoring can support monitoring and detection of the potential spread of GM horticultural species such as GM apple trees, as it also covers traffic and transport areas, settlements and settlement greens, hedges as well as forests. With regard to the monitoring of GM fish and GM microalgae, ecosystem monitoring can monitor a potential degradation of habitat quality. However, as the recorded parameters are of a general nature, conclusions on cause–effect relationships due to novel GMO applications will hardly be possible. 

The forest monitoring of the National Heritage can also be useful to monitor the possible occurrence and spread of GM apple trees in the respective areas.

In addition to the biodiversity monitoring programs, further monitoring programs in Germany should complement the above-mentioned national biodiversity schemes. In particular, the monitoring program according to the Water Framework Directive (WFD, Directive 2000/62/EC) will be important for monitoring the potential effects of GM aquatic species. The WFD monitoring covers a range of different environmental aspects, such as ecological and chemical water conditions, the ecological potential of waters, different protection targets, as well as aquatic flora and fauna, such as benthic invertebrates, fish, and phytoplankton [169]. However, surveying the production of aquaculture activities is not covered by this monitoring scheme. For this purpose, aquaculture statistics would need to provide the relevant data on GM fish use. 

In all biodiversity monitoring programs considered, pest or pathogen (viral or bacterial) indicators are not specifically included. Similar to classical insect-resistant GM crops, the development of resistance in target organisms in disease-resistant GMOs and potential shifts in pest or disease pressure are risks that must be monitored (e.g., for GM applications in orchards). In addition, the specific insect vectors of target pathogens and the disease incidence, as is the case for GM virus applications, need to be surveyed. Some of these will have to be addressed by additional monitoring schemes, such as agricultural monitoring or monitoring by the phytosanitary services of the Federal States in Germany. Monitoring virus dispersal is considered a particular challenge; however, it will be required for novel GM virus applications [88].

## 6. Conclusions

Novel GMO applications will require different monitoring approaches than GM crops used in managed agro-ecosystems. The novel GMO applications discussed here challenge the current implementation of post-market environmental monitoring in the EU. GMO monitoring concepts and methods developed so far primarily address potential effects in agro-ecosystems and are hardly applicable to other types of organisms than annual crop plants and other receiving environments. 

At present, organisms modified by either classical transgenesis or new genomic techniques are covered by the provisions of Directive 2001/18/EC. Thus, in addition to a science-based assessment of risks to human health and the environment, post-market environmental monitoring (PMEM) for the period of authorization is required. The monitoring should take into account the specific risk hypotheses formulated in the risk assessment, but it also needs to consider unexpected and long-term effects. Until now, experience with monitoring GMOs has been limited in the EU. Only one GM crop plant (*Bt* maize) is currently cultivated in Europe with regular monitoring activities. For this GM crop, the focus is on the surveillance of potential resistance development in target organisms of the *Bt* trait. Potential environmental effects in or outside the maize cultivation area are not addressed. In addition, there is no experience with the integration of data from existing biodiversity monitoring in PMEM for *Bt* maize in the EU. Therefore, the suitability of such routine surveillance programs for GMO monitoring is uncertain. With novel GMO applications on the horizon, it is necessary to scrutinize the existing monitoring concepts, methods, and surveillance programs to determine their applicability for detecting potential adverse effects on the environment.

While general monitoring principles and approaches remain applicable for the monitoring of novel GMO applications, the specific monitoring designs, indicators, parameters, and methods must be aligned with the specific GMO application with respect to the specific exposure routes and potentially affected habitats and species. Besides other biodiversity indicators, novel GMO applications may also require the monitoring of non-biodiversity-related aspects, such as pest and pathogen vectors and animal or plant health. Monitoring viral pathogens and insect vector species or the monitoring of aquatic biodiversity are novel requirements that have not been addressed in GMO monitoring yet, both conceptually and practically. Therefore, additional surveillance activities addressing these specific aspects of novel GMO applications will be needed.

The examples of novel types of GMOs in this study also show the necessary extension of the monitoring area beyond agro-ecosystems. Habitats, which have rarely been taken into account so far, such as parks, settlement areas, forests, and specifically aquatic environments, have to be included in PMEM. The existing GMO monitoring concepts and guidelines focus on potential effects on agro-ecosystems and are mainly useful for novel GMO applications in the agricultural or horticultural context. If other receiving environments are concerned, adaptations are needed on a case-by-case basis, particularly in terms of the area for monitoring and the selection of indicators. 

Due to the ability of novel types of GMOs to spread and persist for longer time periods (e.g., GM fruit trees), the monitoring requirements differ not only on a spatial but also on a temporal scale from classical GM crops. The ability of long-lived organisms such as GM freshwater fish to spread actively or passively and to reproduce in the wild requires not only spatial but also temporal expansion of the monitoring period beyond the authorization period. 

We therefore highlight the importance of existing nationwide biodiversity monitoring programs in complementing GMO monitoring in the future. These can cover the monitoring of potential harm to biodiversity and the environment beyond agro-ecosystems and can provide reference areas and reference data for the interpretation of monitoring results. Moreover, they are important for the observation and surveillance of unexpected and long-term effects, as required by the regulatory provisions of the current legal framework. 

In the context of PMEM, the availability of a detection method for the respective GMOs and the establishment of a register indicating the release locations or production facilities of GMO applications will facilitate the identification of pathways into natural habitats and the environment, thus ensuring appropriate and focused post-market environmental monitoring. Challenges addressing the detection, identification, and traceability of plants obtained by NGT have recently been reviewed elsewhere [170,171] and may also apply to other novel types of GMOs. Recently, two EU-funded projects have been launched to address the detection challenges for products developed by new genomic techniques [172,173].

Applications based on new genomic techniques (NGT) can target a broad range of organisms and traits that were previously not achievable with classical transgenesis. With these novel types of GMOs, an increase in the spatial and temporal scale of GMO releases is likely. As for other autonomously reproducing living organisms, such as non-native species, GMOs can spread, persist, and survive unintendedly in different environmental compartments, with unknown implications for biodiversity, nature conservation, and ecosystem services. This lack of predictability and the resulting uncertainties require the development and integration of completely new monitoring methods to account for the specificities of certain novel applications, such as GM viruses and GM microorganisms. The importance of post-release environmental monitoring as a cornerstone in the European Union’s regulatory framework for GMOs should therefore be acknowledged when discussing potential legal changes in the regulation of certain NGT plants at the EU level. 

## Figures and Tables

**Table 1:** Potential environmental effects and novel monitoring requirements for GM applications in fruit orchards (based on Annex II of Directive 2001/18/EC).

Potential Effects	Novel Monitoring Requirements
Occurrence and spread of GM trees or GM viruses outside cultivation areas (including outcrossing of GM trait into wild relatives)	Occurrence of the GM traits in feral/wild apples and wild relatives (*Malus* sp., *Pyrus* sp., *Sorbus* sp.), including species of conservation concern Virus dispersal (incl. vectors) in orchards and host plants if occurring in (semi-) natural habitats
Changes in pathogen/disease spectrum e.g., due to resistance formation of the target pathogen	Disease incidence in orchards (including relevant vector species)
Changes in the infectivity, pathogenicity of the GM virus (in comparison with native strains)	Virus biology in orchards
Intensification of GM tree cultivation including changes in pesticide use	Cultivation intensity and pesticide use of GM fruit trees in orchards
Effects on non-target organisms and the biodiversity in and in close proximity to fruit orchards	Occurrence of non-target organisms and indicators for biodiversity (e.g., vegetation, pollinators, beneficial insects, birds) in orchards and surroundings

**Table 2:** Potential environmental effects and novel monitoring requirements for GM freshwater fish (based on Annex II of Directive 2001/18/EC).

Potential Effects	Novel Monitoring Requirements
Occurrence and spread of GM freshwater fish to and in natural habitats Hybridization of GM freshwater fish with wild relatives, including native and autochthonous species	Frequency of occurrence of GM fish and hybridization with non-GM fish in diverse freshwater ecosystems
Effects on species composition in aquatic food webs (fish, invertebrates)	Aquatic food webs and biodiversity in relevant aquatic habitats
Displacement of native (autochthonous) fish species	Frequency of occurrence of selected fish taxa
Decrease in water quality	Water quality
Spread of parasites and diseases to native (autochthonous) populations in natural habitats	Fish disease incidence and parasites
Intensification of inland aquaculture production	Intensity of freshwater fish production

**Table 3:** Potential environmental effects and novel monitoring requirements for GM microalgae (based on Annex II of Directive 2001/18/EC).

Potential Effects	Novel Monitoring Requirements
Occurrence and spread of GM microalgae in natural aquatic habitats Transfer of GM trait to wild-type algae	Frequency of occurrence of GM microalgae in aquatic habitats
Shifts in composition of microalgae communities	Microalgae communities (e.g., baseline condition, keynote species)
Effects on biodiversity in aquatic communities (different trophic levels)	Aquatic food webs and biodiversity in relevant aquatic habitats
Decrease in water quality and of ecosystem services	Water quality (incl. algal blooms) and ecosystem services

**Table 4:** Novel monitoring requirements for GM applications in orchards and coverage by existing biodiversity monitoring programs, NTO = non-target organisms; - = not covered.

GM Applications in Orchards	Biodiversity Monitoring Program
Spread of GM fruit trees	FFH, Ecosystem, National Heritage
Effects on NTO, biodiversity	Bird, FFH, Insect, HNV
Resistance development, disease incidence, viral pathogenicity	-
Intensification of production, pesticide use	-

**Table 5:** Novel monitoring requirements for GM freshwater species and coverage by existing biodiversity monitoring programs. FFH = Fauna and Flora Habitat, WFD = Water Framework Directive. - = not covered.

GM Freshwater Species	Biodiversity Monitoring Program
Spread of GM microalgae/GM fish	FFH, WFD
Aquatic biodiversity and food webs	Bird, FFH, Insect, WFD
Water quality (incl. algal blooms)	Ecosystem, WFD
Fish disease incidence and parasites	-
Intensification of fish production	-

## Data Availability

No new data were created or analyzed in this study. Data sharing is not applicable to this article.
